# The Crosstalk Between the Anterior Hypothalamus and the Locus Coeruleus During Wakefulness Is Associated with Low-Frequency Oscillations Power During Sleep

**DOI:** 10.3390/clockssleep7040053

**Published:** 2025-09-26

**Authors:** Nasrin Mortazavi, Puneet Talwar, Ekaterina Koshmanova, Roya Sharifpour, Elise Beckers, Ilenia Paparella, Fermin Balda, Christine Bastin, Fabienne Collette, Laurent Lamalle, Christophe Phillips, Mikhail Zubkov, Gilles Vandewalle

**Affiliations:** 1 GIGA-Institute, CRC-Human Imaging, University of Liège, 4000 Liège, Belgium; ptalwar@uliege.be (P.T.); ekaterina.koshmanova@chuliege.be (E.K.); roya.sharifpour@ulb.be (R.S.); ebeckers@uliege.be (E.B.); ipaparella@uliege.be (I.P.); fbalda@uliege.be (F.B.); christine.bastin@uliege.be (C.B.); f.collette@uliege.be (F.C.); laurent.lamalle@uliege.be (L.L.); c.phillips@uliege.be (C.P.); m.zubkov@uliege.be (M.Z.); 2 PsyNCog, University of Liège, 4000 Liège, Belgium

**Keywords:** locus coeruleus, hypothalamus, sleep, 7 Tesla functional magnetic resonance imaging, aging

## Abstract

Animal studies show that sleep regulation depends on subcortical networks, but whether the connectivity between subcortical areas contributes to human sleep variability remains unclear. We investigated whether the effective connectivity between the LC and hypothalamic subparts during wakefulness relates to sleep electrophysiology. Thirty-three younger (~22 y, 27 women) and 18 late middle-aged (~61 y, 14 women) healthy individuals underwent 7-Tesla functional MRI during wakefulness to assess LC–hypothalamus effective connectivity. Additionally, sleep EEG was recorded at night in the lab to examine the relationships between effective connectivity measures and REM sleep theta energy as well as sigma power prior to REM. Connectivity analyses revealed strong mutual positive influences between the LC and both the anterior–superior and posterior hypothalamus, consistent with animal studies. Aging was negatively associated with the connectivity from the anterior–superior hypothalamus (including the preoptic area) to the LC. In late middle-aged adults, but not younger adults, stronger effective connectivity from the anterior–superior hypothalamus to the LC was associated with lower REM theta energy. This association extended to other low-frequency bands during REM and NREM sleep. These findings highlight the age-dependent modulation of LC–hypothalamus interactions and their potential roles in sleep regulation, providing new insights into neural mechanisms underlying age-related sleep changes.

## 1. Introduction

Sleep and wakefulness regulation and the fine-tuning of vigilance state are mostly regulated by a circuit of subcortical nuclei located in the basal forebrain, thalamus, hypothalamus and brainstem as part of the ascending activating system [1,2,3]. The locus coeruleus (LC), located in the brainstem, is the main source of norepinephrine (NE) in the brain and sends widespread monosynaptic projections to nearly all brain regions [4,5]. Research in animal models showed that its activity must decrease for transitioning from wakefulness to sleep. During sleep, the LC further shapes the switch between slow wave sleep (SWS) and rapid eye movement sleep (REMS) as well as some of the microstructure elements of sleep [6]. Investigations in humans indicated that the degeneration of the LC is likely driving part of the alteration in sleep–wake regulation commonly found in healthy and pathological aging [7]. Although indirect measures of the activity of the LC did not differ between healthy participants and insomnia patients [8], LC hyperactivity could contribute to a state of hyper arousal during wakefulness while it would either reduce REMS occurrence or REM bout stability during sleep, two phenomena associated with insomnia disorder [9]. In line with the latter hypothesis, we recently reported that a balanced activity of the LC during wakefulness is associated with a more intense REMS, as indexed by the overnight energy over the most typical oscillatory mode of REMS (theta oscillations) [10,11]. The activity of the LC during wakefulness was further associated with the sigma power immediately preceding REM sleep episode [11], a sleep feature that has been causally linked with the activity of the LC in animal models [12].

Research in animal models also established that several nuclei of the hypothalamus are key elements of the circuit regulating sleep and wakefulness. The posterior part of the hypothalamus includes the lateral hypothalamus (LH) and the tuberomammillary nucleus (TMN) which produce orexin and histamine, respectively, which are two neuromodulators stimulating wakefulness, while the LH further produces melanin-concentrating hormone (MCH) which is considered to stabilize sleep and REMS in particular [3]. The LH is further known to exert an excitatory influence over the LC through glutamatergic afferents [13]. The superior part of the anterior hypothalamus includes preoptic nuclei inhibiting the nuclei of the ascending activating system, including the LC, notably through the production of gamma-aminobutyric acid (GABA) and favor sleep initiation [2]. The inferior part of the anterior hypothalamus further includes the main circadian clock—in the suprachiasmatic nucleus—which organizes sleep and wakefulness in time of the 24 h light–dark cycle mostly through vasoactive intestinal peptide (VIP) and GABA [14], and notably through indirect projection to the LC [15]. Similarly to the LC, the nuclei of the hypothalamus undergo age-related changes, which can disrupt their regulatory role in sleep, potentially contributing to alterations of sleep patterns in healthy and pathological aging [16].

As for the LC, most of our understanding of the roles of the hypothalamus nuclei in sleep and wakefulness regulation arises from animal studies such that translations to human beings are needed if one wants to develop efficient interventions geared toward the neuromodulator systems underlying vigilance state in healthy individuals and patients. Whether the crosstalk between the LC and the nuclei of the hypothalamus underlies variability in sleep electrophysiology and its age-related changes has not yet been investigated.

Similarly to our initial reports of an association between LC activity and REM sleep, we hypothesized that the crosstalk between the LC and the hypothalamus during wakefulness would reflect a trait that would also be present during sleep [10,11]. By using high-field 7 Tesla functional magnetic resonance imaging (7T fMRI) and stochastic Dynamic Causal Modeling (DCM) during wakefulness, we aimed to capture biologically plausible connectivity patterns that reflect enduring functional relationships between sleep-regulatory regions. These interactions may not be tied to task-specific modulation but could represent stable neuromodulatory dynamics relevant to sleep physiology. We used the same 7T fMRI dataset as our initial studies [10,11] to test whether the effective connectivity between the LC and subparts of the hypothalamus would be related to REM theta energy and sigma power prior to REM episodes (i.e., the sleep parameters related to wakefulness LC activity in our previous reports [10,11]). Based on functional and anatomical studies in animals, we anticipated that the connectivity between the LC and the anterior–superior, anterior–inferior or the posterior–lateral subpart of the hypothalamus would be associated with the quality of REMS. We further anticipated that the associations would be affected by aging.

## 2. Results

**Figure 1:**
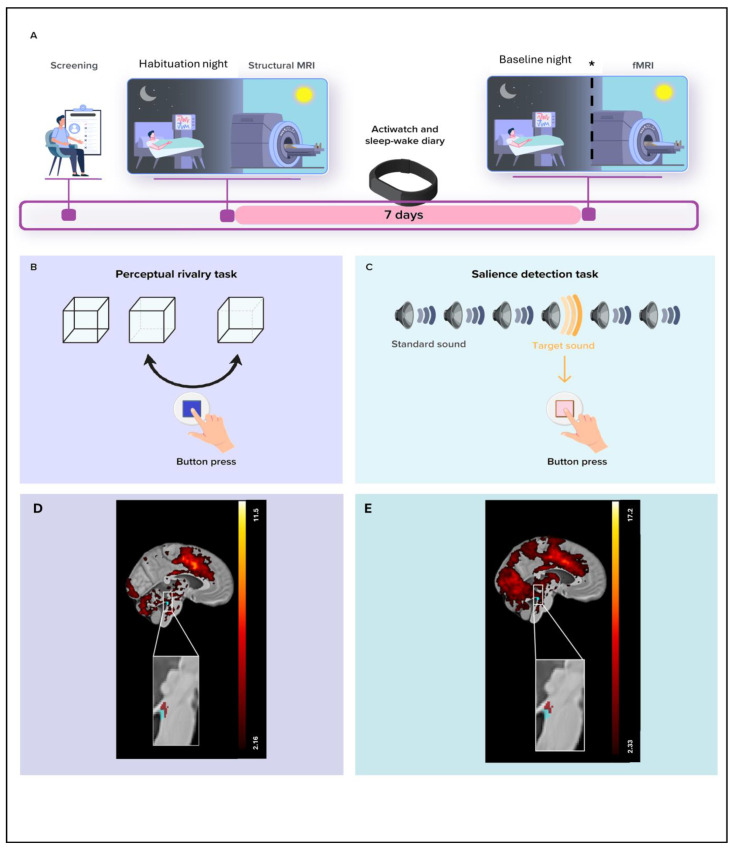
Overview of the study protocol. (**A**) After screening, participants completed an in-lab screening (i.e., habituation night) under polysomnography to minimize the effect of the novel environment for the subsequent baseline night and to exclude volunteers with sleep disorders. They further completed a structural 7T MRI session including a whole-brain structural MRI and a LC-specific sequence. After 7 nights of regular sleep–wake time at home, which was confirmed by actigraphy data and/or sleep–wake diary, participants came to the lab three hours before their sleep time and were maintained in dim light (<10 lux) until sleep time. Participants’ habitual baseline sleep was recorded overnight in-lab under EEG to extract our main sleep features of interest. All participants underwent an fMRI session approximately 3 h after wake-up time (following ≥45 min in dim light—<10 lux), during which they completed the visual–perceptual rivalry task. * In late middle-aged participants, sMRI and fMRI sessions were completed about 1.25 y later than the habituation and baseline nights (mean ± SD: 15.5 ± 5.3 months). Prior to the fMRI session, late middle-aged participants slept regularly for 1 week (verified with a sleep diary). Late middle-aged participants were maintained in dim light (<10 lux) for 45 min before the fMRI scanning. The sleep recording procedure was the same for both younger and late middle-aged participants. (**B**) The visual–perceptual rivalry task consisted of watching a 3D Necker cube, which can be perceived in two different orientations (blue arrow), for 10 blocks of 1 min separated by 10 s of screen-center cross fixation (total duration ~12 min). Participants reported switches in perception through a button press. (**C**) The auditory salience detection task consisted of an oddball paradigm requiring button-press reports on the perception of rare deviant target tones (20% occurrence) within a stream of frequent tones (total duration ~10 min). (**D**) Whole-brain and LC responses to the perceptual switches during the visual–perceptual rivalry task. [MNI coordinates: (−4, −37, −21 mm)]. The image at the top shows the whole-brain results using significance for a threshold of *p* < 0.05 FDR-corrected (t > 2.16) over the group average brain structural image coregistered to the MNI space. The inset at the bottom shows the LC probabilistic template (blue) created based on individual LC masks and the significant activation detected within this mask (red). The legend shows the t-values associated with color maps. (**E**) Whole-brain and LC responses to the target sound during the auditory salience detection task [MNI coordinates: (−4, −34, −21 mm)]. The image at the top shows the whole-brain results using significance for a threshold of *p* < 0.05 FDR-corrected (t > 2.33) over the group average brain structural image coregistered to the MNI space. The inset at the bottom shows the LC probabilistic template (blue) and the significant activation detected within this mask (red). The legend shows the t-values associated with color maps. This figure is adapted from [11].

Fifty-one healthy individuals participated in the study, including 33 young adults (18–30 y) and 18 late middle-aged adults (50–70 y) (Table 1). Participants first underwent an in-lab polysomnography night to rule out sleep disorders, followed by a baseline night of EEG sleep recording, out of which we computed our sleep metrics of interest, i.e., REM theta energy and sigma power prior REM sleep. Approximately three hours after waking from the baseline night (or following a comparable regular sleep period for late middle-aged participants), they completed a 7T fMRI session (Figure 1A) including a visual–perceptual rivalry task (Figure 1B) and an auditory salience detection task (Figure 1C). These tasks were chosen because they were expected to engage the LC [17,18], and, as shown in our earlier work, both indeed elicited robust LC responses [10,11] (Figure 1D,E). In the present work, we extended our focus to the hypothalamus using DCM which can estimate directional influences between regions (effective connectivity).

We applied an automated segmentation algorithm to divide the hypothalamus into five subparts—anterior–inferior, anterior–superior, posterior, inferior tubular, and superior tubular—each comprising several nuclei [19]. We selected the hypothalamus subpart to include in our connectivity analyses in each task (see Materials and Methods). Specifically, we ran two GLMMs for each task, using REM theta energy and sigma power prior to REM episodes as dependent variables and hypothalamus subpart activity as the predictor. Only subparts showing at least a statistical trend (*p* < 0.1) with one of these sleep metrics were retained for the subsequent DCM analyses, ensuring that connectivity analyses focused on hypothalamic regions most likely to be functionally relevant to our sleep measures of interest.

**Figure 2:**
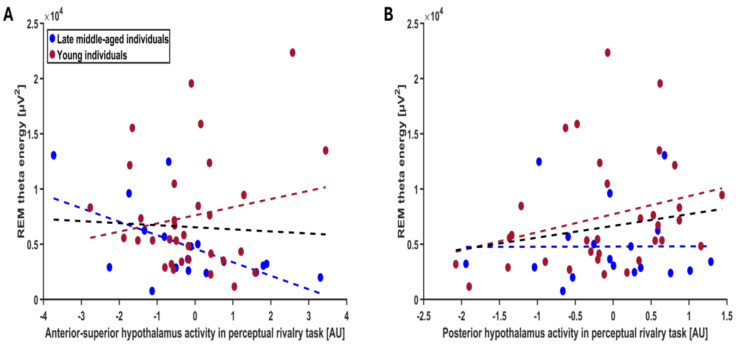
Association between REM theta energy and the anterior–superior and posterior hypothalamus activity during the perceptual rivalry task. The GLMM yielded a statistical trend for the hypothalamus activity by hypothalamus subpart interaction (*p* = 0.08), and post hoc analyses led to a statistical trend for a negative association with the anterior–superior hypothalamus activity (*p* = 0.07) (**A**) and a nominal significance for a positive association with the posterior hypothalamus activity (*p* = 0.05) (**B**) Simple regression lines are used for a visual display and do not substitute the GLMM outputs (Table 2). The black line represents the regression irrespective of age groups (young + late middle-aged adults). The red line represents the regression in young individuals, and the blue line represents the regression in late–middle aged individuals. Non-meaningful associations (i.e., no statistical significance or trend) between REM theta energy or sigma power prior to REMS and the activity of all the subparts of hypothalamus during the perceptual rivalry task and salience detection task are displayed on Figures S1–S4.

Considering the perceptual rivalry task, we found a statistical trend for an association between REM theta energy and the interaction between hypothalamus activity and hypothalamus subparts (f = 2.05; *p* = 0.08; Table 2). Post hoc contrasts revealed that REM theta energy shows a negative statistical trend with the response of the anterior–superior hypothalamus (t = −1.81; *p* = 0.07) and a positive nominally significant association with the response of the posterior hypothalamus (t = 1.90; *p* = 0.05; Figure 2A,B). The other subparts did not yield statistical association with REM theta energy (t < −0.02; *p* > 0.18) (Figure S1, Table S1). The GLMM seeking potential associations between sigma power prior to REM and the activity of the different hypothalamus subparts during the perceptual rivalry task did not reveal any statistical meaningful associations (Figure S2, Table 2, *p* = 0.86). Considering the auditory salience detection task, the GLMMs including REMS theta energy or sigma power prior to REMS as dependent variable did not reveal any statistically significant association with hypothalamus subpart activity (Figures S3 and S4, Table 2).

**Figure 3:**
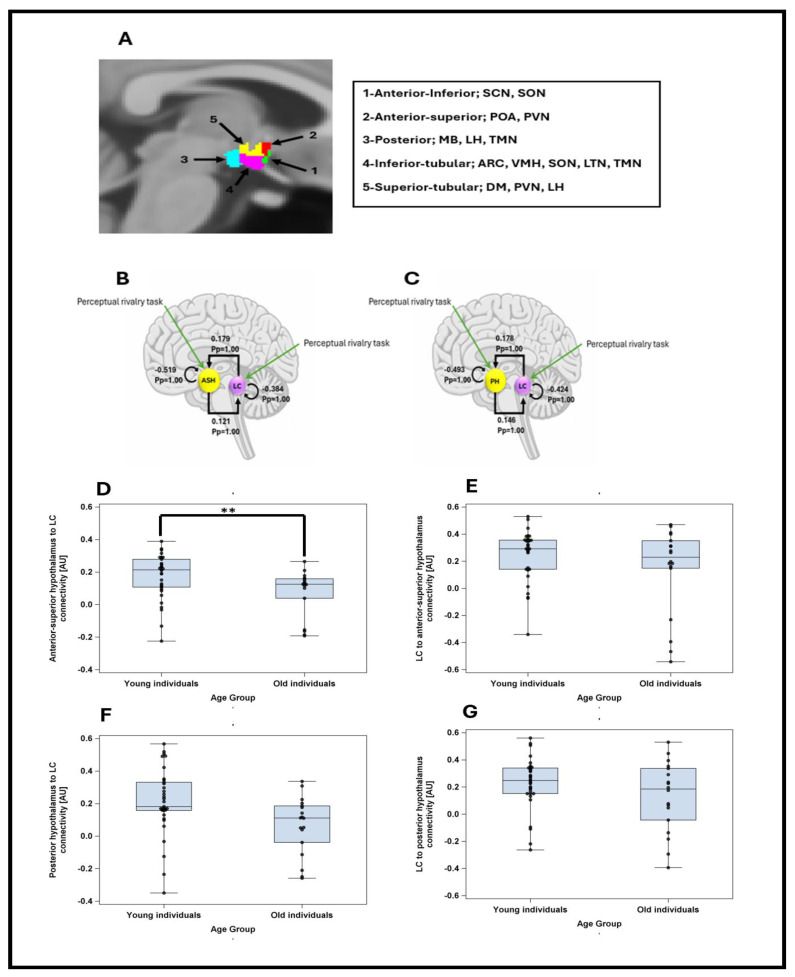
(**A**) Segmentation of the hypothalamus in five subparts in a representative participant. The nuclei encompassed by the different subparts are indicated in the right inset—according to [19]. ARC: Arcuate nucleus; DM: dorsomedial nucleus; LH: lateral hypothalamus; LTN: lateral tubular nucleus; MB: mamillary body; POA: preoptic area; PVN: paraventricular nucleus; SCN: suprachiasmatic nucleus; SON: supraoptic nucleus; TMN: tuberomammillary nucleus; VMN: ventromedial nucleus. (**B**) The DCM model, which included intrinsic connections between anterior–superior hypothalamus (ASH) and locus coeruleus (LC), along with self-feedback gain control connections for both regions. Task inputs were further considered to reach both regions. The DCM analysis showed that there was very strong evidence (Pp = 1.0) for reciprocal positive influence between LC and ASH as well as self-inhibition in both LC and ASH. (**C**) The DCM model, which included intrinsic connections between posterior hypothalamus (PH) and locus coeruleus (LC), along with self-feedback gain control connections for both regions. Task inputs were further considered to reach both regions. The DCM analysis showed that there was very strong evidence (Pp = 1.0) for reciprocal positive influence between LC and PH as well as self-inhibition in both LC and PH. (**D**) Anterior–superior hypothalamus to LC connectivity in young and late middle-aged groups. Younger individuals had significantly higher connectivity than late middle-aged individuals (t = −2.27; *p* = 0.027). (**E**) LC to anterior–superior hypothalamus connectivity in young and late middle-aged groups. Connectivity strengths were not significantly different between two age groups (t = −1.32; *p* = 0.193). (**F**) Posterior hypothalamus to LC connectivity in young and late middle-aged groups. Connectivity strengths were not significantly different between two age groups (t = −1.49; *p* = 0.142). (**G**) LC to posterior hypothalamus connectivity in young and late middle-aged groups. Connectivity strengths were not significantly different between two age groups (t = −0.72; *p* = 0473). **: *p* < 0.05.

Therefore, the DCM analysis concentrated on the connectivity between the LC and the anterior–superior or the posterior subparts of the hypothalamus during the perceptual rivalry task and sought relationship with REM theta energy, as it was the sole sleep metric that yielded a statistical trend with the activity of these hypothalamus subparts (Figure 3A illustrates the nuclei included in each subpart). As the task did not elicit a strong activation in the anterior–superior or the posterior subparts of the hypothalamus, we used stochastic DCM [20], which can estimate directional influences between regions even without robust task-evoked responses. This method enables the detection of latent network dynamics and meaningful connectivity patterns that do not depend on pronounced BOLD activation in every region.

The DCM analyses first showed that there was strong evidence for reciprocal positive influence between LC and both subparts of hypothalamus (Pp = 1.0), confirming that the connectivity anticipated based on animal model [2], can be detected in humans using a stochastic DCM approach (Figure 3B,C). We computed separate GLMMs to determine whether each of the four connectivity parameters between the LC and hypothalamus nuclei (as dependent variable) varied between age groups, controlling for sex and TIV. The models yielded a significant difference between age groups only for the connectivity from the anterior–superior hypothalamus to the LC (t = −2.27; *p* = 0.027), with late middle-aged individuals showing a reduced excitatory connectivity parameters compared with the younger individuals (Figure 3D–G, Supplementary Table S2 for full statistical output of the GLMMs including each connectivity parameters).

**Figure 4:**
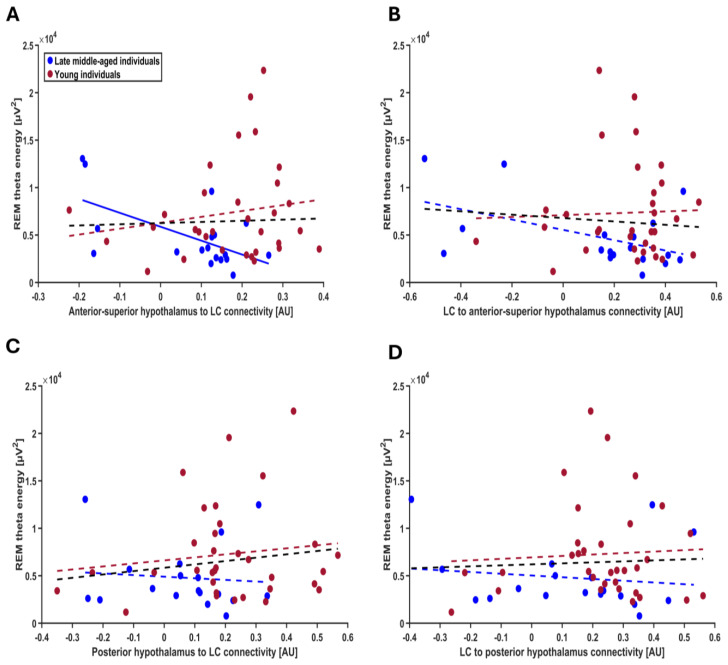
Association between REM theta energy and the connectivity metrics between the LC and the hypothalamus nuclei. (**A**) Connectivity from the anterior–superior hypothalamus to the LC. The GLMM yielded a significant age group by connectivity interaction (*p* = 0.026), and post hoc analyses led to a significant association for the late middle-aged (*p* = 0.042) but not the young group (*p* = 0.294). After excluding one putative outlier in TIV (≥4 SD), the connectivity-by-age-group interaction became more robust (t = 2.37; *p* = 0.022). (**B**) Connectivity from the LC to the anterior–superior hypothalamus. The GLMM did not yield a significant age group by connectivity interaction. (**C**) Connectivity from posterior hypothalamus to the LC. The GLMM did not show a significant age group by connectivity interaction. (**D**) Connectivity from the LC to the posterior hypothalamus. The GLMM did not show a significant age group by connectivity interaction. Simple regression lines are used for a visual display and do not substitute the GLMM outputs (Table 3). The black line represents the regression irrespective of age groups. Solid and dashed regression lines represent significant and non-significant outputs of the GLMM, respectively.

To address our primary aim to test for potential associations between each connectivity parameter and REMS theta energy, we computed four separate GLMMs with REMS theta energy as dependent variable and the interaction between connectivity parameters and age group as independent variable, controlling for sex, total intracranial volume (TIV) and total sleep time. The model including the connectivity from anterior–superior subpart of hypothalamus to LC yielded a significant connectivity-by-age-group interaction (t = 2.30; *p* = 0.026), on top of a main effect of TST, while the other covariates were not significant (Table 3). Post hoc contrasts highlighted that in the late middle-aged individuals, higher excitatory connectivity (i.e., positive connectivity values) was associated with lower REM theta energy, while stronger inhibitory connectivity (i.e., negative connectivity values) was associated with higher REM theta energy (t = −2.09; *p* = 0.042). No such association was detected in the younger group (t = 1.06; *p* = 0.294) (Figure 4A). In addition, after removing a participant, who had an outlier TIV value (SD = 4.078), the connectivity-by-age-group interaction became more robust (t = 2.37; *p* = 0.022; i.e., below the multiple comparison correction *p* < 0.025 threshold) with post hoc contrasts identically showing the association between higher connectivity and lower REM theta energy in the late middle-aged group (t = −2.12, *p* = 0.039), but not in the younger group (t = 1.15, *p* = 0.255). We then considered the connectivity from LC to anterior–superior subpart of hypothalamus, but GLMM did not yield significant association with REM theta energy (Table 3; Figure 4B). Likewise, when we considered the connectivity between the posterior hypothalamus subpart and the LC (i.e., both to and from the LC), GLMMs did not lead to any significant association with REM theta energy (Table 3; Figure 4C,D). In summary, the only connectivity parameters showing age-related difference is also the only one showing an age-dependent association with REMS theta energy.

**Figure 5:**
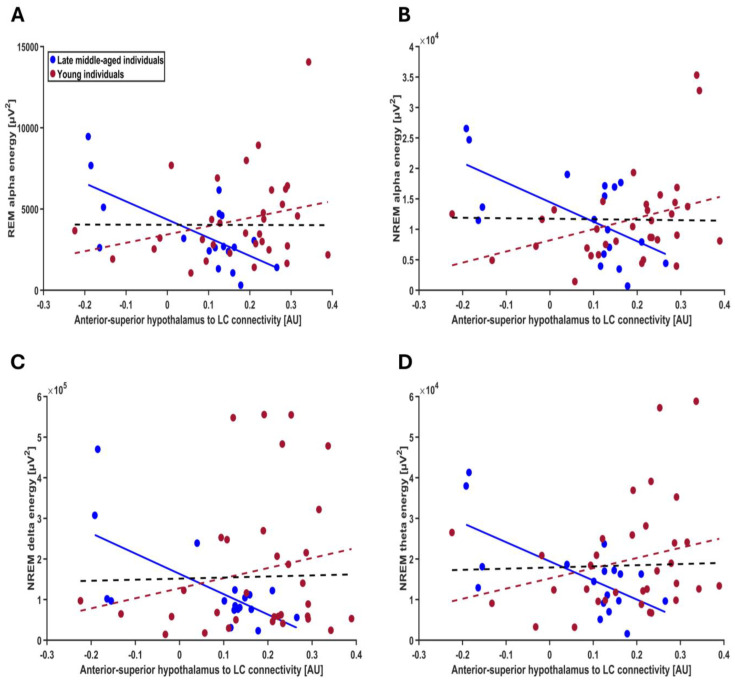
Exploratory association between other frequency bands of the EEG during both REM and NREM sleep and the connectivity from the anterior–superior hypothalamus to the LC (**A**) REM alpha energy; the GLMM yielded a significant age group by connectivity interaction (*p* = 0.002), and post hoc analyses led to a significant association for the late middle-aged (*p* = 0.007) but not the younger group (*p* = 0.108). (**B**) NREM alpha energy; the GLMM yielded a significant age group by connectivity interaction (*p* = 0.002), and post hoc analyses led to a significant association for the late middle-aged (*p* = 0.0008) but not the younger group (*p* = 0.099). (**C**) NREM delta energy; the GLMM yielded a significant age group by connectivity interaction (*p* = 0.015), and post hoc analyses led to a significant association for the late middle-aged (*p* = 0.047) but not the younger group (*p* = 0.142). (**D**) NREM theta energy; the GLMM yielded a significant age group by connectivity interaction (*p* = 0.005), and post hoc analyses led to a significant association for the late middle-aged (*p* = 0.023) but not the younger group (*p* = 0.085). Simple regression lines are used for a visual display and do not substitute the GLMM outputs (Table 4). The black line represents the regression irrespective of age groups. Solid and dashed regression lines represent significant and non-significant outputs of the GLMM, respectively. Non-significant associations between the energy of other frequency bands and the connectivity from anterior–superior hypothalamus to the LC are displayed on Figure S5.

In the next steps, we verified the specificity of our finding for REM theta energy and turned toward the other frequency bands of the EEG during both REM and NREM and found that although association did not extend to all frequency bands, they were not restricted to REM theta energy. Separate GLMMs found that connectivity from the anterior–superior subpart of the hypothalamus to the LC by age-group interaction was significantly associated with several lower-frequency bands of both REM and NREM: interaction was significant for alpha energy in REMS (t = 3.21; *p* = 0.0025), delta energy in NREMS (t = 2.51; *p* = 0.016), theta energy in NREMS (t = 2.91; *p* = 0.006) and alpha energy in NREM (t = 3.17; *p* = 0.0028) but not for delta, sigma and beta energy in REM and sigma and beta energy in NREM; each time we observed a significant negative association between connectivity and frequency band energy in the late middle-aged group (REM alpha: t = −2.79; *p* = 0.008; NREM delta: t = −2.04; *p* = 0.047; NREM theta: t = −2.34; *p* = 0.024; alpha NREM: t = −2.75; *p* = 0.009) but not younger group (t ≤ 1.76; *p* ≥ 0.085 for the four frequency bands) (Figure 5A–D, Table 4; non-significant associations between exploratory sleep metrics and the connectivity from anterior–superior hypothalamus to LC are presented in Table S3).

## 3. Discussion

The interactions between subcortical structures regulating sleep are not fully established in humans. Here, we used 7 Tesla fMRI to capture the crosstalk between the hypothalamus and the LC and related it to the electrophysiology of REM sleep. We presumed that the connectivity between subparts of the hypothalamus and the LC during wakefulness would reflect in part their connectivity during sleep and would therefore be relevant to sleep physiology. We provide evidence that lower REM theta energy is associated with higher effective connectivity from the anterior–superior hypothalamus, which encompasses the preoptic area, to LC in the late middle-aged individuals of our sample. The association was not specific to REM theta energy and extended to other (but not all) lower-frequency bands of both REM and NREM sleep. These findings constitute an original investigation of how a small network of subcortical areas may take part in sleep regulation in humans and provide novel insights into the changes in sleep taking place over the healthy lifespan.

The tasks included in the protocol were geared toward ensuring a reliable recruitment of the LC [10,11] while they did not strongly recruit the hypothalamus. We therefore used an effective connectivity approach that was geared to such cases (i.e., stochastic DCM). We isolated candidate hypothalamus subparts that could be included in our connectivity models by seeking at least weak associations with our two sleep metrics of interest. We found weak indications that the activity of the posterior and anterior–superior subparts of the hypothalamus during the perceptual rivalry task were associated with REM theta energy, but not with sigma power prior to REM episodes. These indications were used to guide our connectivity analyses and will not be interpreted further although they warrant future investigations. We stress that the fact that we focused on specific subparts of the hypothalamus does not preclude the connectivity between the LC and other subparts of the hypothalamus that would be assessed in other contexts to be related to sleep electrophysiology, e.g., the anterior–inferior subpart encompassing the SCN [15] (i.e., if activity was assessed using different cognitive tasks, resting state fMRI, or a different vigilance state, etc.). Likewise, the connectivity of the LC with other parts of the brain in the perceptual rivalry as well as in the salience detection task may turn out to be related to sleep physiology in other analyses. The fact that we obtained high evidence (Pp = 1) that the posterior and anterior–superior subparts were part of a network with the LC demonstrates that one can grasp a meaningful part of the complex interplays between the LC and nuclei of the hypothalamus in vivo in humans following our approach. There was indeed no guarantee that the network we constructed would be related to the fMRI signal we extracted for the hypothalamus and the LC. Our findings suggest that the mutual influence of the anterior–superior and posterior subparts of hypothalamus on the LC repeatedly demonstrated in animal [21,22] can be isolated in humans. This important proof-of-concept paves the way for future investigations that could be built around other regions and more complex networks.

Following the extraction of the connectivity metrics from the two networks, respectively, composed of the posterior subpart of the hypothalamus and the LC and of the anterior–superior subparts of the hypothalamus and the LC, we find only one metric of the latter networks to be associated with REM theta energy. The anterior–superior subpart included not only the preoptic area, key to sleep regulation, but also the paraventricular nucleus (PVN) [19], which is typically related to food intake, energy balance [23] and vegetative regulation [24]. Hence, we posit that the associations we observed were mostly driven by the preoptic area, which contains the ventrolateral (VLPO), lateral (LPO) and median (MPO) preoptic areas, which have all been involved in sleep regulation [2,25,26]. The neurons of the preoptic area promote sleep onset and sleep maintenance by inhibitory GABAergic modulation of multiple arousal systems such as LC [21,22]. The inhibitory action of the VLPO exerted on LC is considered as a requirement for sleep onset [3].

Among the four connectivity parameters of the two DCM models, we find that only the connectivity from the anterior–superior subparts of the hypothalamus to the LC decreases in the late middle-aged compared with the younger individuals. We further find that the stronger the excitatory connectivity from the anterior–superior hypothalamus to the LC during wakefulness (indicated by positive connectivity values), the lower REM theta energy in late middle-aged individuals, and the stronger the inhibitory connectivity from the anterior–superior hypothalamus to the LC during wakefulness (indicated by negative connectivity values), the higher REM theta energy in late middle-aged individuals. Theta oscillations consist of the most typical oscillatory mode of REM sleep. They are considered to be cortical correlates of the hippocampus ripple waves occurring during sleep and related to the memory function of REM sleep [27]. We interpret these as a reflection of REM sleep intensity. We previously reported that a larger expression of LC responses during the same task during wakefulness was associated with a better expression of REM sleep theta oscillations [11]. Our current finding may therefore indicate that a stronger connection between the preoptic nuclei and the LC prevents the LC from favoring REM sleep in late middle-aged individuals. The connectivity between the preoptic area and the LC, at least during wakefulness and potentially also during sleep, would decrease with aging and the extent of this decrease would contribute to REM sleep variability among late middle-aged individuals and also, potentially, to the decreased expression of REM associated with aging. These potential actions may be related to the overall alteration in the balance of the neural circuits previously reported in aging [28]. They may also be linked to recent studies in rodents reporting that the preoptic nuclei regulates LC activity and prevent LC over reactivity that could become detrimental for the expression of REM sleep [29,30]. We find that the larger inhibitory connectivity is associated with the larger REMS theta expression. How our findings captured during wakefulness in the diurnal human species fit with the reports in animals will require further investigation, including using MRI recordings during sleep.

Interestingly, we found that the associations between anterior–superior hypothalamus to LC connectivity and neural oscillations extend beyond the theta energy in REMS. In late middle-aged individuals, its negative correlations with alpha energy in REMS and NREMS as well as delta and theta energy in NREMS suggest a potential broader role for this connectivity in influencing sleep electrophysiology. Delta energy in NREMS is recognized as a marker of sleep need as well as sleep maintenance, quality, and restorative processes [31]. Higher theta energy in NREMS is also linked to memory reactivation and memory consolidation [32]. In addition, alpha energy during REMS and NREMS is related to better cognitive performance as it is shown that individuals with cognitive impairment have lower alpha energy in REMS and NREMS compared to healthy individuals [33]. Consistent with our main finding involving REM theta energy, the observed negative association between the energy of these frequency bands and the strength of preoptic hypothalamus to LC connectivity in late middle-aged adults may reduce the beneficial effects of these oscillatory dynamics in sleep. It could also represent an adaptive mechanism in late middle-aged individuals to prevent LC overreaction. In any case, it shows that the connectivity from the anterior–superior subpart of the hypothalamus to the LC is associated with neuronal synchrony over the lower range of the EEG spectral bands during sleep, potentially affecting the mechanism related to this synchrony (e.g., memory consolidation, sleep homeostasis). This could contribute to the previous findings on the aging brain’s adaptive modifications in homeostatic sleep control [34].

As noted earlier [10,11], our study bears limitations. Young participants underwent fMRI scanning the day after their baseline night of sleep, while for the late middle-aged group, there was an approximately one-year interval between the baseline sleep night and the fMRI session. Although sleep changes during the lifetime [35], it tends to remain stable over shorter periods (e.g., a few years) [36]. Therefore, we believe this significant limitation does not fully account for our findings. Additionally, while we posit that brain activity and connectivity in wakefulness partly reflects brain activity and connectivity relevant in sleep, this assumption has not been directly demonstrated. Moreover, despite the extensive data collection involved, the sample size, particularly for the late middle-aged cohort, is relatively small. In addition, our sample was predominantly female, a factor accounted for in our statistical analysis but still limiting the broad generalizability of the results. The absence of middle-aged individuals aged 30 to 50 years may have also obscured more gradual, age-related changes in LC–hypothalamus connectivity and sleep electrophysiology. Finally, although stochastic DCM allows modeling of effective connectivity without strong task-related activation, the observed LC–hypothalamus coupling could reflect baseline rather than task-specific modulation. Future studies should include recording of brain activity during wakefulness during other cognitive tasks and during sleep, they should include large and sex-balanced samples of continuous age ranges.

## 4. Materials and Methods

This study was approved by the faculty–hospital ethics committee of ULiège. All participants provided written informed consent and received financial compensation. The study is part of a larger project that has led to previous publications [10,11,37]. Most of the methods were described in detail in [10,11].

### 4.1. Participants

Fifty-two healthy participants were included in the study. Due to technical issues, one subject was excluded, and the final sample included 51 participants, with 33 healthy young (18–30 y, 27 women) and 18 late middle-aged (50–70 y, 14 women) individuals (Table 1). The exclusion criteria were as follows: history of major neurologic/psychiatric diseases or stroke; recent history of depression/anxiety; sleep disorders; medication affecting the central nervous system; smoking, excessive alcohol (>14 units/week) or caffeine (>5 cups/day) consumption; night shift work in the past 6 months; BMI ≤ 18 and ≥29 (for late middle-aged individuals) and ≥25 (for younger individuals). All late middle-aged participants had to show normal performance on the Mattis Dementia Rating Scale (score > 130/144) [38]. Due to a miscalculation at screening, one late middle-aged participant had a BMI of 30.9 and one of the younger participants had a BMI of 28.4. Since their data do not deviate substantially from the rest of the sample, these participants were included in the analyses (including BMI as a covariate in our statistical models did not modify our results).

### 4.2. Protocol

Participants’ sleep was recorded in the lab twice. During the first session, participants completed a night of sleep under polysomnography to screen for sleep abnormalities (apnea hourly index and periodic leg movement > 15; parasomnia or REM behavioral disorder). All participants further underwent a whole-brain structural MRI (sMRI) and a specific acquisition centered on the LC. Participants were then requested to sleep regularly for 7 days before the baseline night during the second session (±30 min from their sleep schedule) based on their preferred schedule (compliance was verified using sleep diaries and wrist actigraphy—Actiwatch and AX3, AXIVITY LTD, Newcastle, UK). The evening before the baseline night, participants first completed questionnaires including Beck depression inventory (BDI) [39], Beck anxiety inventory (BAI) [40], the Pittsburgh sleep quality index (PSQI) [41], Epworth sleepiness scale (ESS) [42] and Horne–Ostberg’s Morningness–Eveningness scale [43] for assessing depression, anxiety, sleep quality, sleepiness and chronotype, respectively. They remained awake for 3 h under dim light (<10 lux) for electrode placement and preparation to sleep prior to the recording of their habitual sleep in darkness under EEG. Approximately 3 h after wake-up time under dim light (<10 lux), participants completed a functional MRI (fMRI) session that included 3 tasks (Figure 1A). This paper is centered on the analyses of the perceptual rivalry task and auditory salience detection task.

Younger participants completed the fMRI session immediately following the baseline night but late middle-aged participants were initially part of a different study [44,45] and completed the sMRI and fMRI recordings in addition to their initial engagement. Late middle-aged participants completed the habituation and baseline night EEG recordings as part of their initial study and the sMRI and fMRI sessions were completed about 1.25 y later as part of the current study (mean ± SD: 15.5 ± 5.3 months). Prior to the fMRI session, late middle-aged participants slept regularly for 1 week (verified with a sleep diary; based on our experience, actigraphy reports and sleep diaries do not deviate substantially in late middle-aged individuals). Late middle-aged participants were maintained in dim light (<10 lux) for 45 min before the fMRI scanning. The sleep recording procedure was the same for both younger and late middle-aged participants. Both groups were allowed to have breakfast before the fMRI session but were instructed to avoid caffeine intake.

### 4.3. Sleep EEG Metrics

Eleven channels were used for the baseline night (F3,z,4; C3,z,4; P3,z,4; O1,2) initially referenced to the left mastoid prior to re-referencing offline to the average of both mastoids (N7000 amplifier, EMBLA, Natus, Middleton, WI, USA). Arousals and artifacts were detected automatically [46] to provide the number of arousals during REM sleep, and excluded from the power spectral density analyses. Only frontal electrodes were considered in the analyses because the frontal region is commonly accepted as most sensitive to sleep homeostasis [47]; focusing on the frontal electrodes may also facilitate interpretation of future large-scale studies using ambulatory EEG, often restricted to frontal electrodes.

Sleep was staged in 30 s epochs using an automatic algorithm (ASEEGA, PHYSIP, Paris) [48] to provide total sleep time (TST). Averaged energy was computed for each 30 min bin, adjusted for the proportion of rejected data. The adjusted values were then summed across REM sleep [49] to provide REM theta energy (overnight cumulated 4.25–8 Hz energy). Energy in the other typical bands of the sleep EEG were computed similarly during both REM and NREM for specificity assessments (Delta band: 0.5–4 Hz; Theta: 4.25–8 Hz; Alpha: 8.25–12 Hz; Sigma: 12.25–16 Hz; Beta: 16.25–30 Hz). Sigma power (12.25–16 Hz) was computed during the 1 min preceding each REM episode (if sleep stage was N2 and N3), as the weighted sum of 4 s artifact-free window (2 s overlap per 30 s epoch), prior to averaging over the number of REM episodes.

### 4.4. Cognitive Tasks

Visual–perceptual rivalry task. The task (~12 min total duration) consisted of watching a 3D Necker cube, which can be perceived in two different orientations (Figure 1B), for 10 blocks of 1 min separated by 10 s of screen-center cross fixation. Participants were instructed to report switches between the two percepts through a button press.

Auditory salience detection task. The task (~10 min total duration) consisted of an oddball paradigm requiring reports on the perception of rare deviant target tones (1000 Hz, 100 ms, 20% of tones) that were pseudo-randomly interleaved within a stream of standard stimuli (500 Hz, 100 ms) through a button press (Figure 1C). The task included 270 stimuli (54 targets).

These tasks were selected because they were thought to activate the LC [17,18]. As we previously reported, both tasks successfully triggered a response of the LC [10,11] (Figure 1D,E).

### 4.5. MRI Data Acquisition, Preprocessing and Univariate Analyses

MRI data were acquired using a MAGNETOM Terra 7T MRI system (Siemens Healthineers, Erlangen, Germany), with a single-channel transmit and 32-receiving channel head coil (1TX/32RX, Nova Medical, Forchheim, Germany). Blood-oxygen-level-dependent (BOLD) fMRI data were acquired using a multi-band (MB) gradient-recalled echo–echo-planar imaging (GRE-EPI) sequence (main parameters: repetition time = 2.340 ms, flip angle = 90°, 86 axial 1.4 mm thick slices, no interslice gap, matrix size = 160 × 160, voxel size = 1.4 × 1.4 × 1.4 mm^3^,MB acceleration factor = 2, GeneRalized Autocalibrating Partial Parallel Acquisition (GRAPPA) acceleration factor = 3). The cardiac pulse and the respiratory movements were recorded concomitantly using, respectively, a pulse oximeter and a breathing belt (Siemens Healthineers). The fMRI acquisition was followed by a dual-echo 2D GRE field mapping sequence to assess B0 magnetic field inhomogeneities with the following parameters: TR = 5.2 ms, TEs = 2.26 ms and 3.28 ms, flip angle (FA) = 15°, bandwidth = 737 Hz/pixel, matrix size = 96 × 128, 96 axial slices with 2 mm thickness, voxel size = 2 × 2 × 2 mm ^3^, acquisition time = 1:38 min.

A Magnetization-Prepared with 2 RApid Gradient Echoes (MP2RAGE) sequence was used to acquire T1 anatomical images: TR = 4300 ms, TE = 1.98 ms, FA = 5°/6°, TI = 940 ms/2830 ms, bandwidth = 240 Hz/pixel, matrix size = 256 × 256, 224 axial 0.75 mm–thick slices, GeneRalized Autocalibrating Partial Parallel Acquisition (GRAPPA) acceleration factor = 3, voxel size = 0.75 × 0.75 × 0.75 mm^3^, acquisition time = 9:03 min [50].

The LC-specific sequence consisted of a 3D high-resolution magnetization transfer–weighted turbo-flash (MT-TFL) sequence with the following parameters [51]: TR = 400 ms, TE = 2.55 ms, FA = 8°, bandwidth = 300 Hz/pixel, matrix size = 480 × 480 × 60, number of averages = 2, turbo factor = 54, magnetization transfer contrast (MTC) pulses = 20, MTC FA = 260°, MTC RF duration = 10,000 μs, MTC inter-RF delay = 4000 μs, MTC offset = 2000 Hz, voxel size = 0.4 × 0.4 × 0.5 mm^3^, acquisition time = 8:13 min. Axial slices were acquired and centered for the acquisitions perpendicularly to the rhomboid fossa (i.e., the floor of the fourth ventricle located on the dorsal surface of the pons) [51].

Functional and anatomical MRI data were preprocessed using SPM12, ANTs and SynthStrip brain extraction tool [52], as fully described previously [10,11]. The preprocessed data were resampled to a 1 mm^3^ resolution. Individual statistical analyses consisted of a general linear model (GLM) including one regressor of interest, consisting of a switch in perception (perceptual rivalry) or target tone (salience detection) modeled as an event (convolved with the canonical hemodynamic response function—HRF). Participant movement parameters, respiration, and heart rate were used as covariates of no interest (physiological data of 4 volunteers were not available and therefore not included in their individual design matrices). The T1 structural whole-brain image was used to extract individual total intracranial volume (TIV) using CAT12 toolbox [53].

Individual LC masks were manually delineated by 2 experts based on LC-specific images (as in [10]) and activity of left LC was extracted in each subpart (as LC responses were more prominent in the left LC in both tasks [10,11]). The different nuclei of the hypothalamus do not offer a lot of contrast in MRI images such that they cannot be segmented using current approaches. We therefore used an automated segmentation algorithm to parcellate the hypothalamus into 5 subparts which encompass several nuclei—anterior–inferior, anterior–superior, posterior, inferior tubular and superior tubular [19] (see Figure 3A for the nuclei deemed to be included in each subpart). We then extracted the activity of these subparts within the left hypothalamus (i.e., mean value over each subpart of hypothalamus) during both tasks for each participant using the REX Toolbox (https://web.mit.edu/swg/software.htm, accessed on 10 January 2024). We selected subparts of the left hypothalamus because we were interested in the effective connectivity between the left hypothalamus and the left LC, where responses were more prominent.

### 4.6. Effective Connectivity Analysis

Our previous analysis of the fMRI data of both tasks indicated only weak hypothalamus activation (i.e., no responses associated with the events of interest were detected even when applying an uncorrected threshold of *p* < 0.001). This does not, in principle, prohibit effective connectivity from being computed and importantly to be associated with other features of interest, such as sleep electrophysiology metrics. To select the hypothalamus subpart to be considered in our connectivity analyses, we reasoned that the activity of a given hypothalamus subpart should at least be weakly associated with our sleep metrics of interest. We therefore determined whether the activity estimate of each subpart—separately for each task—was associated with either REM theta energy or sigma power prior to REM episodes. Left hypothalamus subpart activity was used in GLMM to seek correlation with REM theta energy and sigma power prior to REM sleep episodes (one GLMM per task and per sleep metric) (see Statistic section below). Only the hypothalamus subparts that yielded at least a statistical trend (*p* < 0.1) with either sleep metrics during a given task were considered for effective connectivity analyses.

DCM framework [54], implemented in SPM12, was used to compute the effective connectivity between the LC and each of the selected hypothalamus subparts during the selected task (i.e., the anterior–superior and posterior subparts during the visual–perceptual rivalry task—see Results). BOLD signal time series associated with our event of interest (i.e., perceptual switch) were extracted from individually defined ROIs (i.e., over the LC and hypothalamus subparts masks), based on the individual statistical maps thresholded at *p* < 0.05 uncorrected. During the extraction of the BOLD signal time series, confounding effects such as head movement and physiological noise were regressed out, ensuring the resulting time series better reflected neural activity related to the event. We extracted the first principal component (eigenvariate) of the “adjusted” time series, which represents the time series after regressing out effects of no interest, using the approach outlined by [55].

Stochastic DCM was used because no significant task-related activations were detected in the selected hypothalamus subparts in the group-level whole-brain analysis across all participants (cf. above) [20]. We computed two DCM models based on animal evidence of reciprocal interaction between the LC and the anterior–superior subpart of hypothalamus as well as LC and posterior subpart of hypothalamus [21,22] (i.e., intrinsic connections between the two regions), along with self-feedback gain control connections for LC and hypothalamus subpart (Figure 3B,C). Task inputs were further considered to reach both regions. Time series extracted from individual ROIs were subjected to a first-level DCM analysis, where the model was estimated for each subject. To isolate the connectivity parameters that were contributing to the model and could therefore be used in a GLMM seeking associations with sleep metrics (see below), we performed a Parametric Empirical Bayes (PEB) analysis [56,57] over the first-level DCM parameter estimates. PEB is a hierarchical Bayesian model that evaluates commonalities and differences among subjects in the effective connectivity domain at the group level by performing Bayesian model reduction (BMR), which explores the space of DCM models and leads to a subset of models best explaining the data and Bayesian model averaging (BMA) of the parameters across models weighted by the evidence of each model. Subsequently, as there is no concept of significance in Bayesian analysis, we reported and used in the GLMM, the parameters contributed to the model evidence with a posterior probability (Pp) exceeding 0.90.

### 4.7. Statistics 

GLMMs were performed in SAS 9.4 (SAS Institute, Cary, NC, USA) and were adjusted for the distribution of the dependent variables. Outliers among connectivity and sleep metrics lying beyond four times the standard deviation were removed from the analysis (maximum one data point was removed, the final number of individuals included in each analysis is reported in each table). The first GLMMs were meant to isolate which hypothalamus subparts would be included in DCM. They included the 2 sleep features of interest as dependent variables in each task separately and hypothalamus subpart, hypothalamus activity estimates and age group, including sex, TST and TIV as covariates (i.e., 4 models in total, 1 per sleep metric and task). Our initial models included the three-way interaction between hypothalamus activity, hypothalamus subpart and age group and all three simple two-way interaction terms. Non-significant three-way/two-way interactions were removed from the models based on Bayesian Index Criterion (BIC) for fit quality estimation such that final models only included the hypothalamus activity and hypothalamus subpart interaction. This yielded statistical trends with the activity of the anterior–superior and posterior subparts during the perceptual rivalry task (see Results) for REM theta energy so that DCM included these 2 subparts during the latter task.

The next set of GLMMs tested for associations between connectivity metrics and REM theta energy, again used as dependent variable, and including sex, TST and TIV as covariates. Following the same procedure described in the preceding paragraph, all final models included an interaction term between the connectivity metric and the age group. Semi-partial R^2^ (R^2^*) values were computed to estimate the effect sizes of significant fixed effects and statistical trends in all GLMMs [58]. Significance was determined following the Benjamini–Hochberg procedure for false discovery rate procedure [*p* < 0.025 (for rank 1/2); *p* < 0.05 (for rank 2/2)].

We computed a prior sensitivity analysis to obtain an indication of the minimum detectable effect size in our main analyses given our sample size. According to G*Power 3 (version 3.1.9.4) [59]; taking into account a power of 0.8, an error rate α = 0.025, and a sample size of 51, we could detect medium effect sizes *r* > 0.39 (2-sided; CI: 0.13–0.6; *R*^2^ > 0.15, CI: 0.02–0.36) within a linear multiple-regression framework including 2 tested predictor (connectivity, age group) and 2/3 covariates (sex, TIV, TST where relevant).

## 5. Conclusions

In summary, we show that the connectivity between key subcortical structures for sleep regulation assessed during wakefulness may reflect their crosstalk during sleep and contribute to the variability of sleep electrophysiology. Our main finding includes the dominant oscillatory mode of REM, as a potential reflection of REM intensity and amnesic function. The association is detected in participants aged between 50 and 70 y and beyond REM theta rhythms, suggesting a more prominent impact in the fragile sleep found in aging and on neuronal synchrony over lower frequencies. These results underscore the age-dependent modulation of LC circuitry and its potential implications for sleep regulation and the age-related increase in sleep complaints.

## Figures and Tables

**Table 1:** Characteristics of the study sample.

	Young Individuals (*n* = 33)		Late Middle-Aged Individuals (*n* = 18)		*p*-Value
	Mean	SD	Mean	SD	
**Age (years)**	22.20	3.20	60.88	5.41	**<0.0001**
**BMI (kg/m^2^)**	21.90	2.74	24.75	3.40	**0.0047**
**Education (years)**	14.46	2.31	14.61	2.70	0.85
**Depression level (BDI)**	6.64	4.17	5.33	4.01	0.27
**Anxiety level (BAI)**	4.00	2.92	3.05	3.18	0.30
**TIV (cm^3^)**	1402.1	136.6	1420.5	125.5	0.627
**Daytime sleepiness (ESS)**	6.93	3.90	5.77	3.76	0.30
**Sex (F–M)**	28 F–6 M		12 F–6 M		0.20
**Habitual Subjective sleep quality (PSQI)**	4.79	2.18	3.83	2.22	0.14
**Chronotype** **(Horne–Ostberg’s Morningness–Eveningness)**	43.41	7.22	50.50	7.78	**0.001**
**TST (min)**	454.80	30.97	394.63	48.16	**<0.0001**
**REMS duration**	120.3	21.49	82.46	29.06	**<0.0001**
**Theta energy in REMS (µV^2^)**	7281.5	5061.7	4789.5	3508.2	**0.0449**
**Sigma power prior to REMS (µV^2^)**	15.7615	8.8957	16.0544	6.795	0.896

The *p*-values shown in the table correspond to two-sample *t*-tests except for sex that were compared using a Chi-square test. BMI: Body mass index; BDI: Beck depression inventory; BAI: Beck anxiety inventory; TIV: total intracranial volume; PSQI: Pittsburgh sleep quality index; ESS: Epworth sleepiness scale; TST: total sleep time; REMS: rapid eye movement sleep. See Materials and Methods for references for the questionnaires.

**Table 2:** Associations between the two sleep metrics of interest and the 5 hypothalamus subparts activity during the perceptual rivalry task and the salience detection task.

Task	Sleep Metric (Dependent Variable)	Hypothalamus Activity	Hypothalamus Subpart	Hypothalamus Activity *Hypothalamus Subpart	Age Group	Sex	TIV	Total Sleep Time
perceptual rivalry task	REM Theta energy (N = 51)	F(1,239) = 0.62 *p* = 0.432	F(4,239) = 0.05 *p* = 0.994	F(4,239) = 2.05 ***p =* 0.088** R^2^* = 0.033	F(1,239) = 0.44 *p* = 0.507	F(1,239) = 0.94 *p* = 0.334	F(1,239) = 0.53 *p* = 0.466	F(1,239) = 28.5 ***p* < 0.0001** R^2^* = 0.106
perceptual rivalry task	Sigma power prior to REMS (N = 51)	F(1,239) = 0.58 *p* = 0.446	F(4,239) = 0.00 *p* = 1.00	F(4,239) = 0.32 *p* = 0.864	F(1,239) = 2.74 *p* = 0.098	F(1,239) = 20.19 ***p* < 0.0001** R^2^* = 0.077	F(1,239) = 15.03 ***p* = 0.0001** R^2^* = 0.059	F(1,239) = 1.52 *p* = 0.218
salience detection task	REM Theta energy (N = 51)	F(1,239) = 0.00 *p* = 0.950	F(4,239) = 0.01 *p* = 0.999	F(4,239) = 0.22 *p* = 0.927	F(1,239) = 1.28 *p* = 0.258	F(1,239) = 1.70 *p* = 0.193	F(1,239) = 3.79 *p* = 0.052	F(1,239) = 33.00 ***p* < 0.0001** R^2^* = 0.121
salience detection task	Sigma power prior to REMS (N = 51)	F(1,234) = 5.00 ***p* = 0.026** R^2^* = 0.020	F(4,234) = 0.01 *p* = 0.999	F(4,234) = 0.65 *p* = 0.628	F(1,234) = 3.74 *p* = 0.054	F(1,234) = 24.94 ***p* < 0.0001** R^2^* = 0.096	F(1,234) = 14.62 ***p* = 0.0002** R^2^* = 0.058	F(1,234) = 2.28 *p* = 0.132

Prior to the analysis, we removed the outliers among connectivity and sleep metrics by excluding the samples lying beyond four times the standard deviation (the final number of individuals included in each analysis is reported below each dependent variable). REM: Rapid eye movement; REMS: rapid eye movement sleep.

**Table 3:** Associations between REM theta energy and the connectivity between the anterior–superior hypothalamus and the LC and between the posterior hypothalamus and the LC.

Type of Connectivity	Sleep Metric (Dependent Variable)	Connectivity	Age Group	Connectivity*Age Group	Sex	TIV	Total Sleep Time
From anterior–superior hypothalamus to LC	REM Theta energy (N = 50)	F(1,43) = 1.09 *p* = 0.302	F(1,43) = 0.62 *p* = 0.436	F(1,43) = 5.29 ***p* = 0.026 ^A^** R^2^* = 0.109	F(1,43) = 0.02 *p* = 0.902	F(1,43) = 0.03 *p* = 0.854	F(1,43) = 4.74 ***p* = 0.035** R^2^* = 0.099
From LC to anterior–superior hypothalamus	REM Theta energy (N = 50)	F(1,43) = 0.38 *p* = 0.539	F(1,43) = 0.17 *p* = 0.686	F(1,43) = 2.21 *p* = 0.144	F(1,43) = 0.05 *p* = 0.825	F(1,43) = 0.00 *p* = 0.981	F(1,43) = 4.73 ***p* = 0.035** R^2^* = 0.099
From posterior hypothalamus to LC	REM Theta energy (N = 50)	F(1,43) = 0.00 *p* = 0.994	F(1,43) = 0.09 *p* = 0.765	F(1,43) = 0.98 *p* = 0.326	F(1,43) = 0.58 *p* = 0.451	F(1,43) = 0.12 *p* = 0.726	F(1,43) = 4.77 ***p* = 0.034** R^2^* = 0.099
From LC to posterior hypothalamus	REM Theta energy (N = 50)	F(1,43) = 0.14 *p* = 0.710	F(1,43) = 0.01 *p* = 0.911	F(1,43) = 0.61 *p* = 0.439	F(1,43) = 0.49 *p* = 0.488	F(1,43) = 0.08 *p* = 0.776	F(1,43) = 4.40 ***p* = 0.041** R^2^* = 0.092

Prior to the analysis, we removed the outliers among connectivity and sleep metrics by excluding the samples lying beyond four times the standard deviation (the final number of individuals included in each analysis is reported below each dependent variable). ^A^ *p* = 0.022 and F(1,42) = 5.61 after excluding one putative outlier in TIV (≥4 SD). LC: Locus coeruleus; TIV: total intracranial volume; REM: rapid eye movement.

**Table 4:** Significant associations between exploratory sleep metrics and the connectivity from anterior–superior hypothalamus to LC.

Sleep Metric (Dependent Variable)	Connectivity	Age Group	Connectivity*Age Group	Sex	TIV	Total Sleep Time
REM alpha energy (N = 50)	F(1,43) = 1.74 *p* = 0.194	F(1,43) = 2.92 *p* = 0.094	F(1,43) = 10.29 ***p* = 0.002** R^2^* = 0.193	F(1,43) = 0.04 *p* = 0.851	F(1,43) = 0.10 *p* = 0.757	F(1,43) = 5.43 ***p* = 0.024** R^2^* = 0.112
NREM delta energy (N = 51)	F(1,44) = 0.59 *p* = 0.445	F(1,44) = 2.02 *p* = 0.162	**F(1,44) = 6.31** ***p* = 0.015** R^2^* = 0.125	F(1,44) = 0.13 *p* = 0.722	F(1,44) = 1.30 *p* = 0.260	F(1,44) = 0.22 *p* = 0.642
NREM theta energy (N = 51)	F(1,44) = 0.73 *p* = 0.396	F(1,44) = 2.21 *p* = 0.144	F(1,44) = 8.48 ***p* = 0.005** R^2^* = 0.161	F(1,44) = 0.14 *p* = 0.714	F(1,44) = 0.57 *p* = 0.452	F(1,44) = 1.33 *p* = 0.254
NREM alpha energy (N = 51)	F(1,44) = 0.83 *p* = 0.368	F(1,44) = 4.36 ***p* = 0.042** R^2^* = 0.090	F(1,44) = 10.04 ***p* = 0.002** R^2^* = 0.185	F(1,44) = 0.09 *p* = 0.765	F(1,44) = 0.00 *p* = 0.973	F(1,44) = 0.37 *p* = 0.546

Prior to the analysis, we removed the outliers among connectivity and sleep metrics by excluding the samples lying beyond four times the standard deviation (the final number of individuals included in each analysis is reported below each dependent variable). The table only includes results with *p* < 0.05; non-significant association are reported in Supplementary Table S3. *p*-values survive correction for multiple comparisons across the nine exploratory tests using the false discovery rate (FDR). LC: Locus coeruleus; TIV: total intracranial volume; REM: rapid eye movement; NREM: non-rapid eye movement.

## Data Availability

The processed data and analysis scripts supporting the results included in this manuscript are openly available via the following open repository: https://gitlab.uliege.be/CyclotronResearchCentre/Public/fasst/lc-hypothalamus-connectivity (accessed on 19 September 2025). The raw data are available on request from the corresponding author due to the potential for subject re-identification and the large volume of data, which prevent open sharing. Access requests will be subject to evaluation by the local Research Ethics Board and will require the signing of a data transfer agreement (DTA).

## Supplementary Materials

The following supporting information can be downloaded at https://www.mdpi.com/article/10.3390/clockssleep7040053/s1. Table S1: Post hoc contrast on the associations between REMS theta energy and hypothalamus subparts activity estimated via the visual perceptual rivalry task; Table S2: Associations between age and the connectivity between the anterior-superior hypothalamus and the LC as well as between the posterior hypothalamus and the LC; Table S3: Non-significant associations between exploratory sleep metrics and the connectivity from anterior-superior hypothalamus to LC; Figure S1: Non-significant associations between hypothalamus subparts activity estimates during the perceptual rivalry task and REM theta energy; Figure S2: Non-significant associations between hypothalamus subparts activity estimates during the perceptual rivalry task and sigma power prior to REMS; Figure S3: Non-significant associations between hypothalamus subparts activity estimates during the salience detection task and REM theta energy; Figure S4: Non-significant associations between hypothalamus subparts activity estimates during the salience detection task and sigma power prior to REMS; Figure S5: Non-significant associations between sleep metrics of interest and the connectivity from anterior-superior hypothalamus to LC to test the specificity.
