# Supplementary material for: The Crosstalk Between the Anterior Hypothalamus and the Locus Coeruleus During Wakefulness Is Associated with Low-Frequency Oscillations Power During Sleep
Source: Clocks Sleep. 2025 Sep 26;7(4):53. doi: 10.3390/clockssleep7040053 (PMC12551093; doi:10.3390/clockssleep7040053)
Supplement: Supplementary file 1 [file clockssleep-07-00053-s001.zip › clockssleep-3787925-supplementary.pdf]

ONLINE SUPPLEMENTARY MAERIAL

The Crosstalk Between the Anterior Hypothalamus and the Locus Coeruleus During Wakefulness Is Associated with Low- Frequency Oscillations Power During Sleep

Nasrin Mortazavi, MSc,[1*] Puneet Talwar, PhD,[1] Ekaterina Koshmanova, PhD, [1] Roya Sharifpour, PhD, [1] Elise Beckers, PhD, [1] Ilenia Paparella, MSc, [1] Fermin Balda, MSc, [1] Christine Bastin, PhD, [1,2] Fabienne Collette, PhD, [1,2] Laurent Lamalle, PhD, [1] Christophe Phillips, PhD,[1] Mikhail Zubkov, PhD, [1] and Gilles Vandewalle, PhD [1*]

[1] GIGA-Institute, CRC-Human Imaging, University of Liège, Liège 4000, Belgium.

[2] PsyNCog, University of Liège, Liège 4000, Belgium

*Corresponding authors: Nasrin Mortazavi and Gilles Vandewalle, GIGA-CRC-Human Imaging, Bâtiment B30, 8 Allée du Six Août, University of Liège-Sart Tilman, 4000 Liège, Belgium.

nasrin.mortazavi@uliege.be

Supplementary Table S1. Post hoc contrast on the associations between REMS theta energy and hypothalamus subparts activity estimated via the visual perceptual rivalry task.

Hypothalamus subpart	Estimate	DF	t value	P
**Interior-inferior hypothalamus**	-0.001	239	-0.02	0.983
**Anterior-superior hypothalamus**	-0.121	239	-1.81	***0.071***
**Posterior hypothalamus**	0.194	239	1.90	***0.058***
**Inferior-tubular hypothalamus**	-0.116	239	-1.15	0.252
**Superior-tubular hypothalamus**	-0.104	239	-1.32	0.187

Supplementary Table S2. Associations between age and the connectivity between the anterior-superior hypothalamus and the LC as well as between the posterior hypothalamus and the LC.

Type of connectivity	Age	Sex	TIV
From anterior-superior hypothalamus to LC	F(1,47)=5.17 **P=0.027** **R^{2*}=0.099**	F(1,47)=0.69 P=0.410	F(1,47)=0.07 P=0.799
From LC to anterior-superior hypothalamus	F(1,47)=1.74 P=0.199	F(1,47)=0.25 P=0.617	F(1,47)=0.00 P=0.962
From posterior hypothalamus to LC	F(1,47)=2.23 P=0.142	F(1,47)=0.40 P=0.529	F(1,47)=0.23 P=0.633
From LC to posterior hypothalamus	F(1,47)=0.52 P=0.473	F(1,47)=1.33 P=0.254	F(1,47)=0.06 P=0.812

LC: locus coeruleus; TIV: total intracranial volume.

Supplementary Table S3. Non-significant associations between exploratory sleep metrics and the connectivity from anterior-superior hypothalamus to LC.

Sleep metric (dependent variable)	connectivity	Age group	connectivity*age group	Sex	TIV	Total sleep time
REM delta energy (N=51)	F(1,44)=0.49<br>P=0.487	F(1,44)=0.66<br>P=0.420	F(1,44)=4.11<br>P=0.048*<br>R^{2*}=0.085	F(1,44)=0.11<br>P=0.745	F(1,44)=0.13<br>P=0.718	F(1,44)=0.86<br>P=0.358
REM sigma energy (N=51)	F(1,44)=0.00<br>P=0.963	F(1,44)=0.83<br>P=0.366	F(1,44)=0.07<br>P=0.795	F(1,44)=0.46<br>P=0.503	F(1,44)=0.26<br>P=0.611	F(1,44)=6.08<br>P=0.017<br>R^{2*}=0.121
REM beta energy (N=50)	F(1,43)=0.15<br>P=0.703	F(1,43)=0.11<br>P=0.743	F(1,43)=0.82<br>P=0.369	F(1,43)=5.50<br>P=0.023<br>R^{2*}=0.113	F(1,43)=4.19<br>P=0.046<br>R^{2*}=0.088	F(1,43)=12.02<br>P=0.001<br>R^{2*}=0.221
NREM sigma energy (N=51)	F(1,44)=0.35<br>P=0.559	F(1,44)=0.00<br>P=0.953	F(1,44)=0.32<br>P=0.575	F(1,44)=0.02<br>P=0.899	F(1,44)=0.13<br>P=0.717	F(1,44)=3.79<br>P=0.058
NREM beta energy (N=51)	F(1,44)=0.17<br>P=0.680	F(1,44)=0.75<br>P=0.390	F(1,44)=0.36<br>P=0.550	F(1,44)=0.06<br>P=0.804	F(1,44)=0.20<br>P=0.657	F(1,44)=4.16<br>P=0.047<br>R^{2*}=0.086

Prior to the analysis, we removed the outliers among connectivity and sleep metrics by excluding the samples lying beyond four times the standard deviation (the final number of individuals included in each analysis is reported below each dependent variable).

*post hoc analysis showed that the significant association between REM delta energy and age group by connectivity interaction considering the connectivity from anterior-superior hypothalamus to LC (p=0.048) is driven by the association between REM delta energy and the difference of connectivity values between two age groups and not each group separately.

LC: locus coeruleus; TIV: total intracranial volume; REM: rapid eye movement; REMS: rapid eye movement sleep; NREM: non-rapid eye movement.

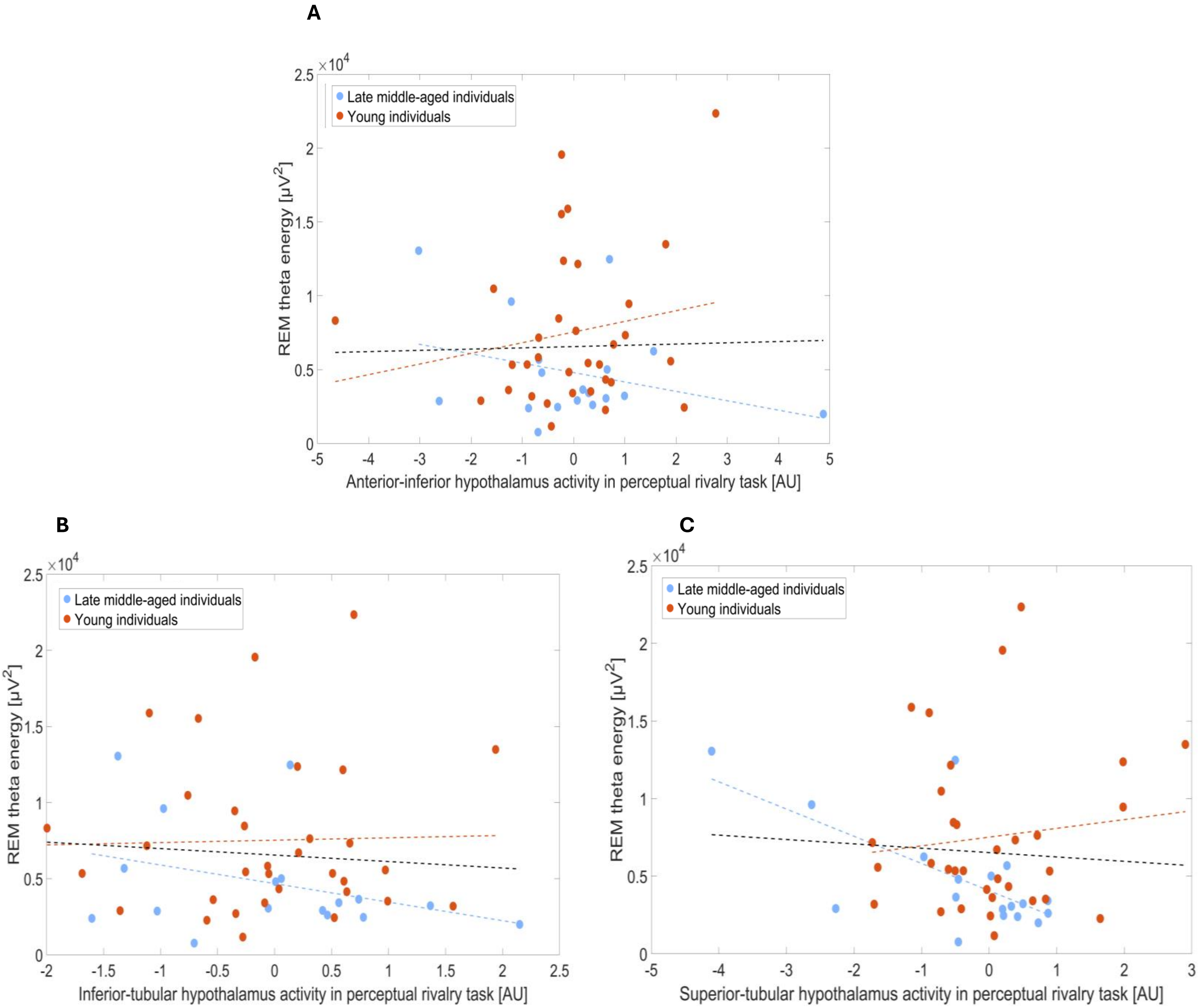
A
B
C
REM theta energy [µV²]
Late middle-aged individuals
Young individuals
Anterior-inferior hypothalamus activity in perceptual rivalry task [AU]
Inferior-tubular hypothalamus activity in perceptual rivalry task [AU]
Superior-tubular hypothalamus activity in perceptual rivalry task [AU]

Supplementary Figure S1. Non-significant associations between hypothalamus subparts activity estimates during the perceptual rivalry task and REM theta energy. (A) Association between the interior-inferior hypothalamus activity estimates during the perceptual rivalry task and REM theta energy. **(B)** Association between the inferior-tubular hypothalamus activity estimates during the perceptual rivalry task and REM theta energy. **(C)** Association between the superior-tubular hypothalamus activity estimates during the perceptual rivalry task and REM theta energy.

Although the GLMM yielded to a statistical trend for the hypothalamus activity by hypothalamus subpart interaction (p=0.8), post hoc analyses did not show a statistical trend for any of these hypothalamus subparts (p>0.18).

Simple regression lines are used for a visual display and do not substitute the GLMM outputs. The black line represents the regression irrespective of age groups (young + old). Dashed regression lines represent non-significant outputs of the GLMM.

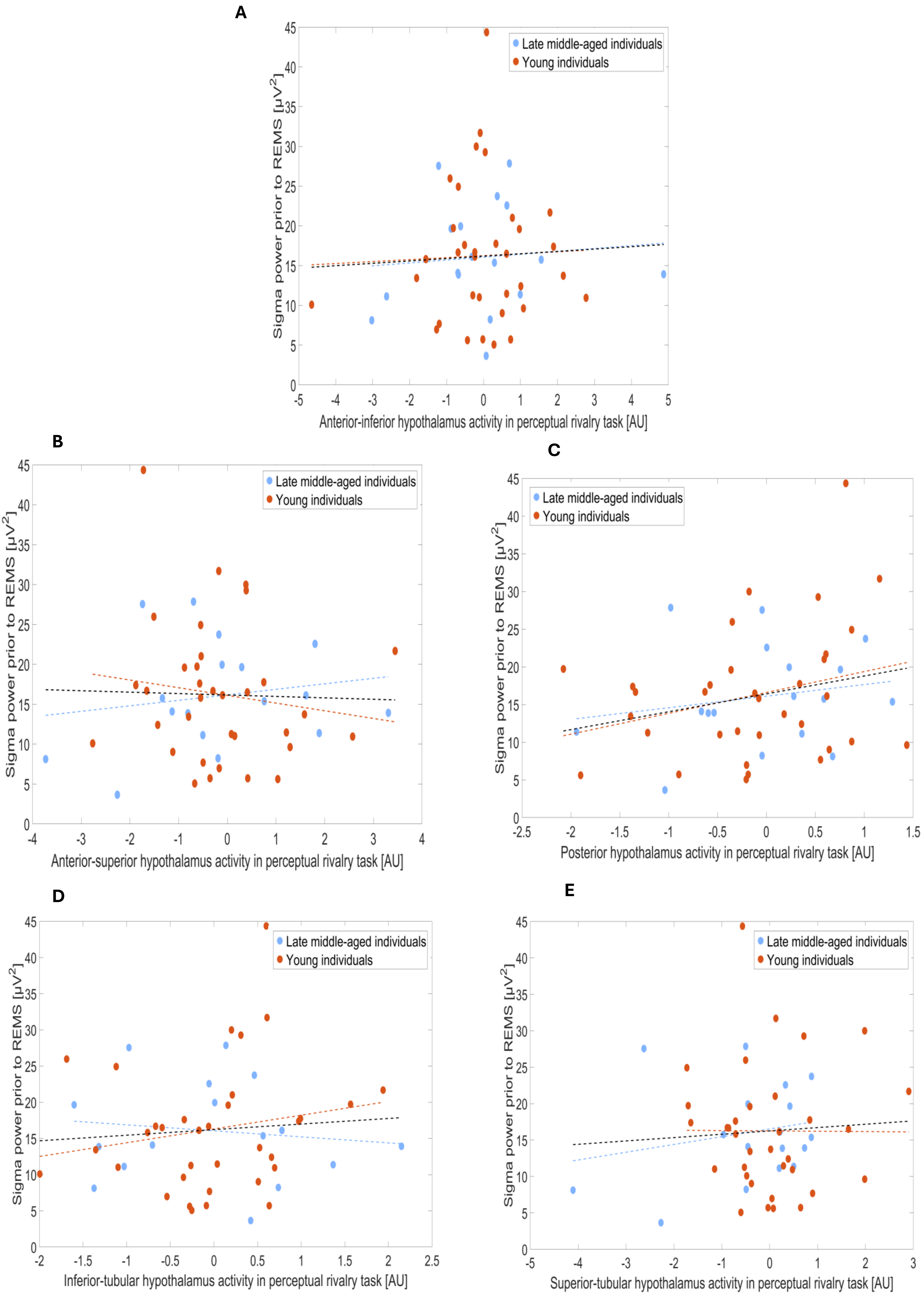
A
B
C
D
E
Late middle-aged individuals
Young individuals
Sigma power prior to REMS [μV²]
Anterior-inferior hypothalamus activity in perceptual rivalry task [AU]
Anterior-superior hypothalamus activity in perceptual rivalry task [AU]
Posterior hypothalamus activity in perceptual rivalry task [AU]
Inferior-tubular hypothalamus activity in perceptual rivalry task [AU]
Superior-tubular hypothalamus activity in perceptual rivalry task [AU]

Supplementary Figure S2. Non-significant associations between hypothalamus subparts activity estimates during the perceptual rivalry task and sigma power prior to REMS. (A) Association between the interior-inferior hypothalamus activity estimates during the perceptual rivalry task and sigma power prior to REMS. **(B)** Association between the interior-superior hypothalamus activity estimates during the perceptual rivalry task and sigma power prior to REMS. **(C)** Association between the posterior hypothalamus activity estimates during the perceptual rivalry task and sigma power prior to REMS. **(D)** Association between the inferior-tubular hypothalamus activity estimates during the perceptual rivalry task and sigma power prior to REMS. **(E)** Association between the superior-tubular hypothalamus activity estimates during the perceptual rivalry task and sigma power prior to REMS.
The GLMM did not yield a statistical trend for the hypothalamus activity by hypothalamus subpart interaction (p=0.8).
Simple regression lines are used for a visual display and do not substitute the GLMM outputs. The black line represents the regression irrespective of age groups (young + old). Dashed regression lines represent non-significant outputs of the GLMM.

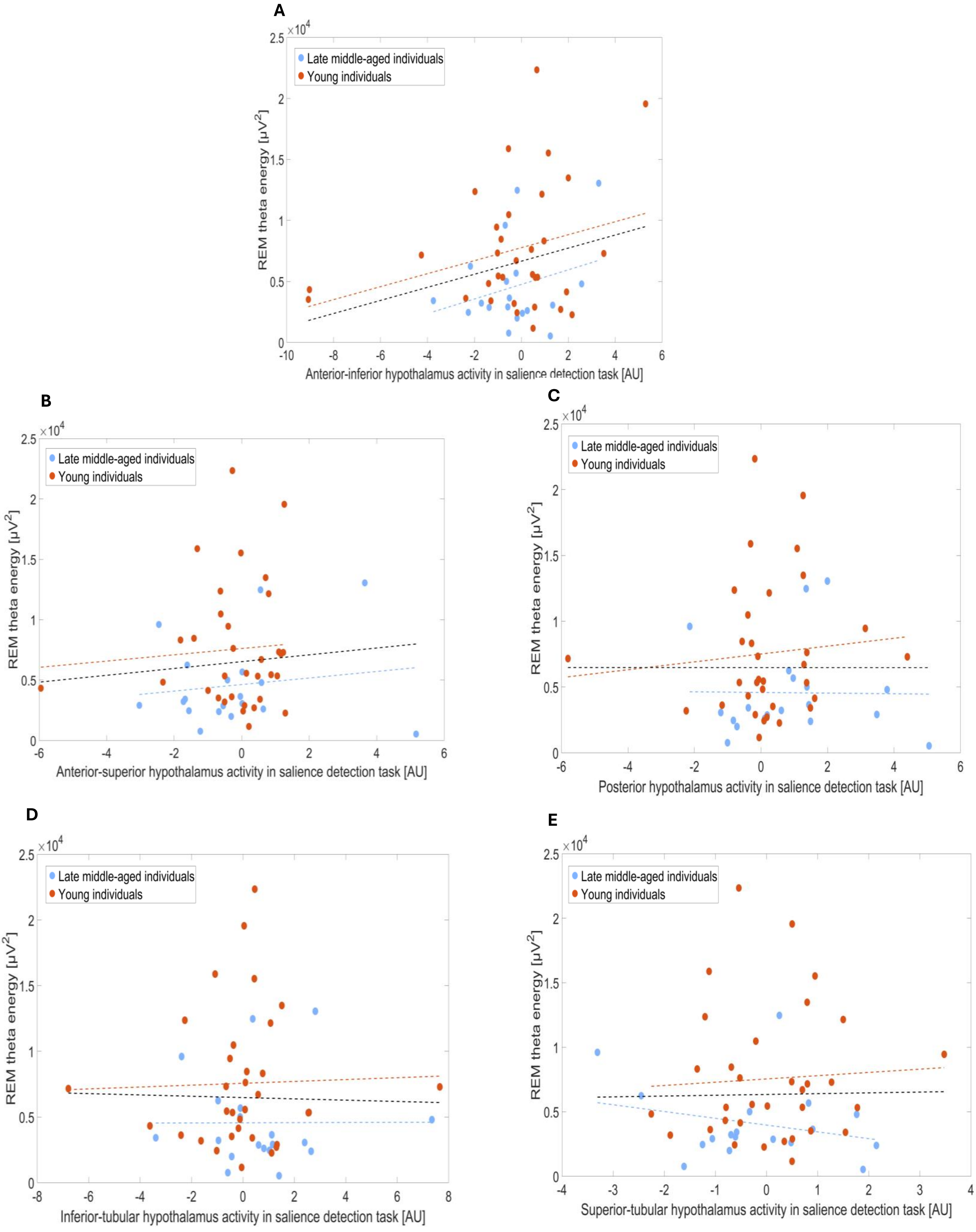
A
B
C
D
E
×10^4
Late middle-aged individuals
Young individuals
REM theta energy [μV^2]
Anterior-inferior hypothalamus activity in salience detection task [AU]
Anterior-superior hypothalamus activity in salience detection task [AU]
Posterior hypothalamus activity in salience detection task [AU]
Inferior-tubular hypothalamus activity in salience detection task [AU]
Superior-tubular hypothalamus activity in salience detection task [AU]

Supplementary Figure S3. Non-significant associations between hypothalamus subparts activity estimates during the salience detection task and REM theta energy. (A) Association between the interior-inferior hypothalamus activity estimates during the salience detection task and REM theta energy. **(B)** Association between the interior-superior hypothalamus activity estimates during the salience detection task and REM theta energy. **(C)** Association between the posterior hypothalamus activity estimates during the salience detection task and REM theta energy. **(D)** Association between the inferior-tubular hypothalamus activity estimates during the salience detection task and REM theta energy. **(E)** Association between the superior-tubular hypothalamus activity estimates during the salience detection task and REM theta energy.

The GLMM did not yield a statistical trend for the hypothalamus activity by hypothalamus subpart interaction (p=0.9).

Simple regression lines are used for a visual display and do not substitute the GLMM outputs. The black line represents the regression irrespective of age groups (young + old). Dashed regression lines represent non-significant outputs of the GLMM.

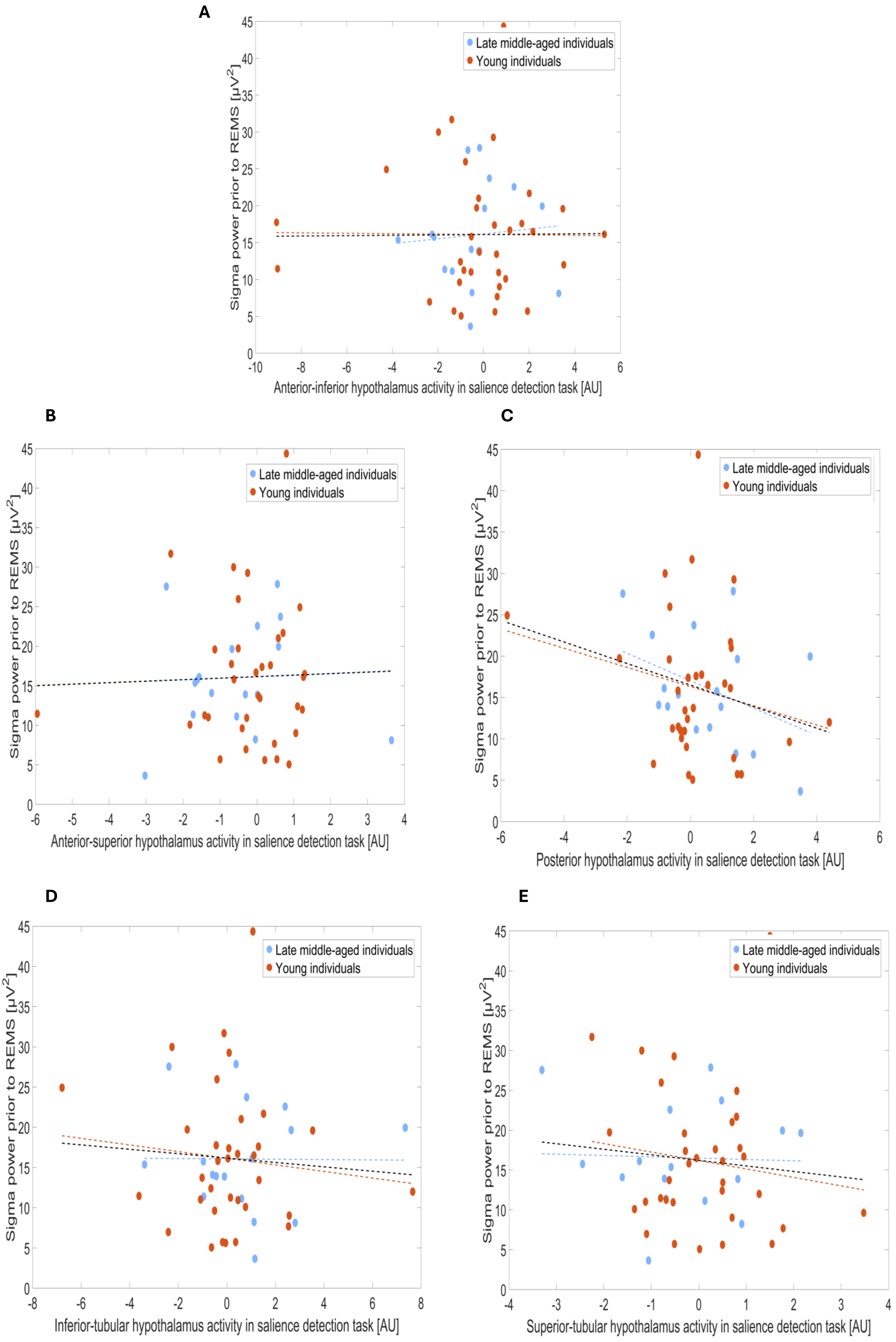

A
Late middle-aged individuals
Young individuals
Sigma power prior to REMS [μV²]
Anterior-inferior hypothalamus activity in salience detection task [AU]
B
Late middle-aged individuals
Young individuals
Sigma power prior to REMS [μV²]
Anterior-superior hypothalamus activity in salience detection task [AU]
C
Late middle-aged individuals
Young individuals
Sigma power prior to REMS [μV²]
Posterior hypothalamus activity in salience detection task [AU]
D
Late middle-aged individuals
Young individuals
Sigma power prior to REMS [μV²]
Inferior-tubular hypothalamus activity in salience detection task [AU]
E
Late middle-aged individuals
Young individuals
Sigma power prior to REMS [μV²]
Superior-tubular hypothalamus activity in salience detection task [AU]

Supplementary Figure S4. Non-significant associations between hypothalamus subparts activity estimates during the salience detection task and sigma power prior to REMS. (A) Association between the interior-inferior hypothalamus activity estimates during the salience detection task and sigma power prior to REMS. **(B)** Association between the interior-superior hypothalamus activity estimates during the salience detection task and sigma power prior to REMS. **(C)** Association between the posterior hypothalamus activity estimates during the salience detection task and sigma power prior to REMS. **(D)** Association between the inferior-tubular hypothalamus activity estimates during the salience detection task and sigma power prior to REMS. **(E)** Association between the superior-tubular hypothalamus activity estimates during the salience detection task and sigma power prior to REMS.

The GLMM did not yield a statistical trend for the hypothalamus activity by hypothalamus subpart interaction (p=0.6).

Simple regression lines are used for a visual display and do not substitute the GLMM outputs. The black line represents the regression irrespective of age groups (young + old). Dashed regression lines represent non-significant outputs of the GLMM.

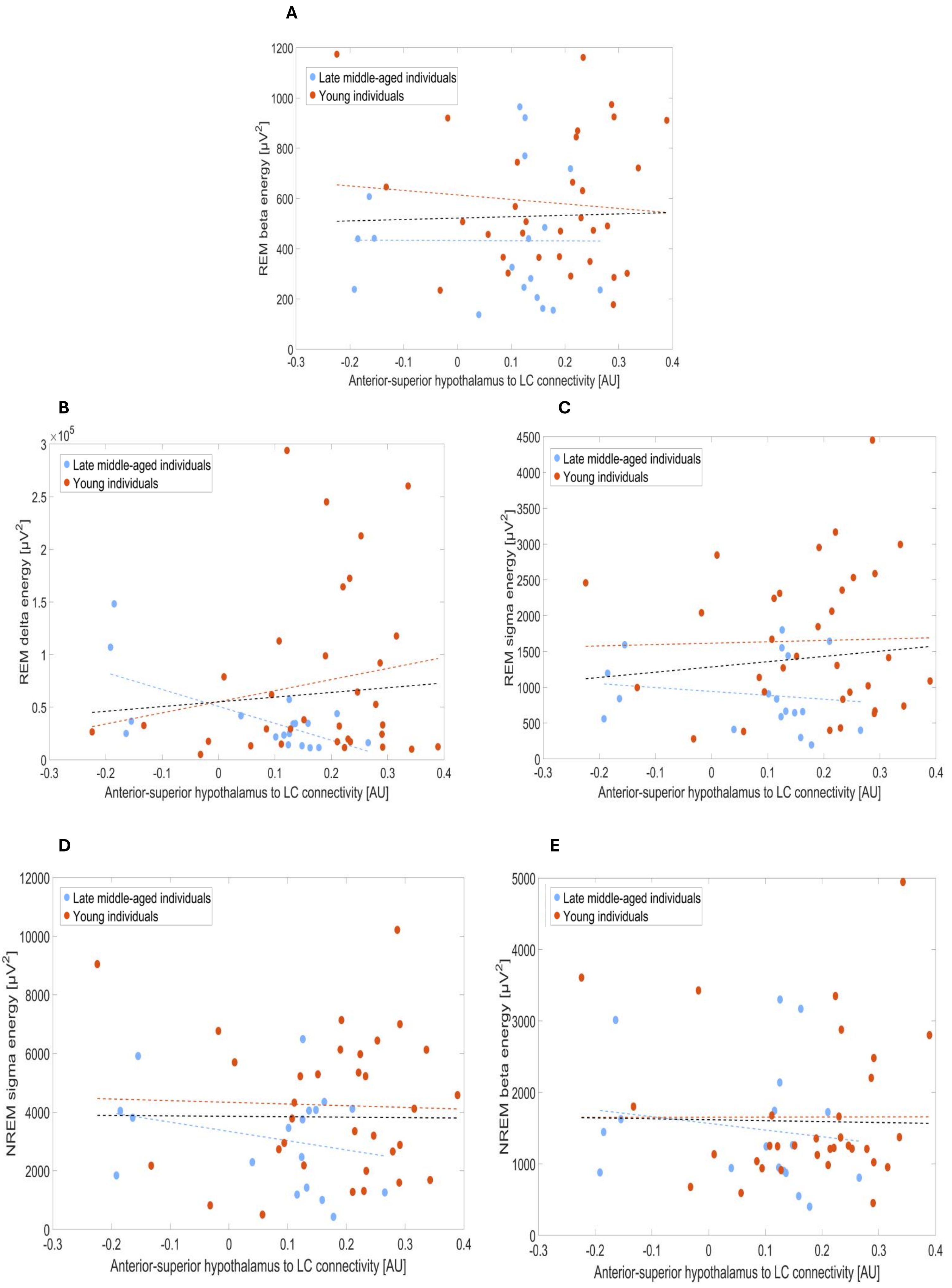
A
Late middle-aged individuals
Young individuals
REM beta energy [μV²]
Anterior-superior hypothalamus to LC connectivity [AU]
0
200
400
600
800
1000
1200
-0.3
-0.2
-0.1
0
0.1
0.2
0.3
0.4
B
×10⁵
Late middle-aged individuals
Young individuals
REM delta energy [μV²]
0
0.5
1
1.5
2
2.5
3
Anterior-superior hypothalamus to LC connectivity [AU]
C
Late middle-aged individuals
Young individuals
REM sigma energy [μV²]
0
500
1000
1500
2000
2500
3000
3500
4000
4500
Anterior-superior hypothalamus to LC connectivity [AU]
D
Late middle-aged individuals
Young individuals
NREM sigma energy [μV²]
0
2000
4000
6000
8000
10000
12000
Anterior-superior hypothalamus to LC connectivity [AU]
E
Late middle-aged individuals
Young individuals
NREM beta energy [μV²]
0
1000
2000
3000
4000
5000
Anterior-superior hypothalamus to LC connectivity [AU]

Supplementary Figure S5. Non-significant associations between sleep metrics of interest and the connectivity from anterior-superior hypothalamus to LC to test the specificity. (A) Association between REM beta energy and the anterior-superior hypothalamus to LC connectivity. **(B)** Association between REM delta energy and the anterior-superior hypothalamus to LC connectivity. **(C)** Association between REM sigma energy and the anterior-superior hypothalamus to LC connectivity. **(D)** Association between NREM sigma energy and the anterior-superior hypothalamus to LC connectivity. **(E)** Association between NREM beta energy and the anterior-superior hypothalamus to LC connectivity.

None of the associations were significant ($p>0.051$).

Simple regression lines are used for a visual display and do not substitute the GLMM outputs. The black line represents the regression irrespective of age groups (young + old). Dashed regression lines represent non-significant outputs of the GLMM.
